# Supplementary figures and images for: Transsynaptic N-Cadherin Adhesion Complexes Control Presynaptic Vesicle and Bulk Endocytosis at Physiological Temperature
Source: Front Cell Neurosci. 2021 Oct 7;15:713693. doi: 10.3389/fncel.2021.713693 (PMC8573734; doi:10.3389/fncel.2021.713693)

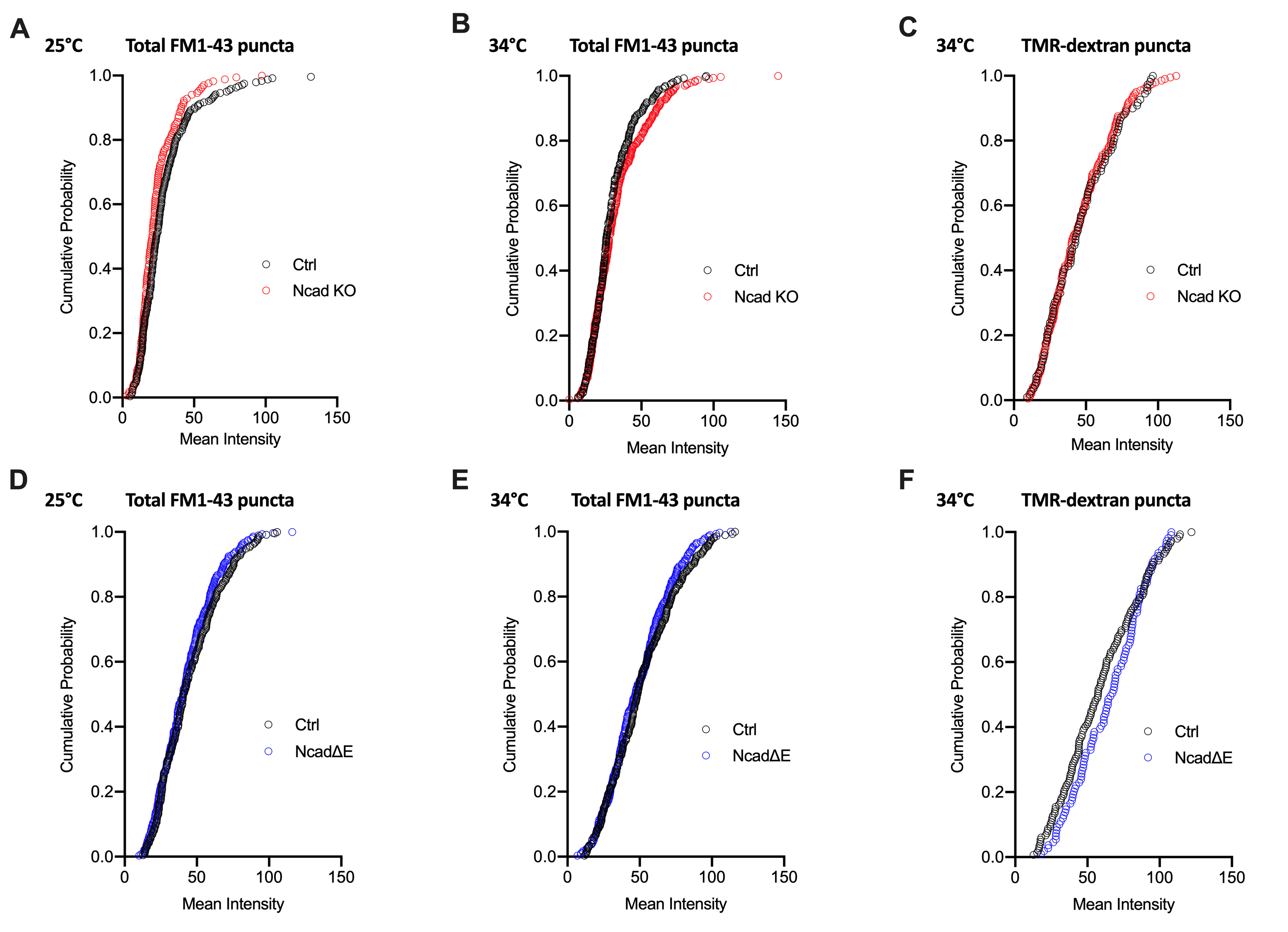

Supplement: Supplementary Figure S1 — Mean fluorescence intensity of FM1–43 and TMR-dextran puncta in N-cadherin knockout neurons and upon inhibition of N-cadherin function. Quantitative analysis of mean pixel fluorescence was performed on thresholded images of FM1–43 and TMR-dextran puncta (see Figure 3 and Figure 4). (A–C) Cumulative probability plots of mean fluorescence intensity of individual puncta from control and N-cadherin knockout neurons. (A) Total FM1–43 puncta at room temperature. (B) Total FM1–43 puncta at near physiological temperature. (C) TMR-dextran puncta at near physiological temperature. (D–F) Cumulative probability plots of mean fluorescence intensity of individual puncta from control and N-cadherinΔE expressing neurons (dominant negative N-cadherin fragment). (D) Total FM1–43 puncta at room temperature. (E) Total FM1–43 puncta at near physiological temperature. (F) TMR-dextran puncta at near physiological temperature. [file Image_1.TIFF]
